# The Use of Methylene Blue in Pilonidal Sinus Surgery Reduces the Risk of Recurrence: A Systematic Review and Meta-Analysis

**DOI:** 10.3390/medicina62020238

**Published:** 2026-01-23

**Authors:** Matthias Maak, Christina Oetzmann von Sochaczewski, Theo Hackmann, Marcel Bonni, Myriam Braun-Münker, Dietrich Doll

**Affiliations:** 1 Department of Surgery, Kreiskrankenhaus St. Anna, 91315 Höchstadt an der Aisch, Germany; 2 Department of Surgery, University Hospital Erlangen, Friedrich-Alexander University Erlangen-Nuremberg, Krankenhausstraße 12, 91054 Erlangen, Germany; 3 Pilonidal Research Group, Vechtaer Research Institute VIFF, Marienstr. 6-8, 49377 Vechta, Germany; 4 Department of Surgery, University Hospital of Bonn, Venusberg-Campus Bonn, 53127 Bonn, Germany; 5 Military Medical Centre Erfurt, Nissaer Weg 10, 99099 Erfurt, Germany; 6 Department of Food Technology, Fulda University of Applied Sciences, Leipziger Straße 123, 36037 Fulda, Germany; 7 Department of Procto-Surgery & Pilonidal Sinus, St. Marienhospital Vechta, Academic Teaching Hospital of the MHH Hannover, Marienstr. 6-8, 49377 Vechta, Germany

**Keywords:** pilonidal sinus, pilonidal sinus disease, PSD, therapy, methylene blue, recurrence, long-term recurrence rate, surgery

## Abstract

*Background and Objectives*: Pilonidal Sinus Disease shows an increasing incidence. Surgical interventions are often necessary; thus, we wanted to analyze whether the usage of methylene blue (MB) for staining the PSD fistulas for better visualization during surgery shows an effect concerning recurrence rates. *Materials and Methods*: A thorough data search and cross-study data synthesis of publications in the time period of 1833 to 2023 lead to a total of 7936 studies focusing on PSD. The data sets were scanned for the eligibility criteria, namely treatment information, follow-up, recurrence data, and the use of MB in surgery. The data were sorted into different surgical treatment groups, and the recurrence rates were analyzed using Kaplan–Meier-survival curves. *Results*: A total of 1192 studies involving 130,677 patients were eligible for analysis. A total of 263 studies were performed with MB (22.1%, *n* = 25,602 patients) and 929 studies without MB (77.9%, *n* = 105,075 patients). Overall, there was a highly significant difference in RR between the group of MB-stained patients and those without (*p* < 0.0001), even if analyzed separately for treatments. Primary open approach, primary midline closure, primary asymmetric closure, and Limberg/Dufourmentel all showed a significantly lower RR when MB was used (*p* < 0.0001). The use of MB in Bascom/Karydakis-Flap showed a statistically significant disadvantage concerning RR (*p* < 0.0001), and Other Flaps showed less RR with the use of MB (*p* = 0.011); both groups showed a notable loss of follow-up data, diminishing the meaning of these *p* values. *Conclusions*: This study shows that the use of MB as a staining technique in PSD surgery may be beneficial regarding recurrence rates. Therefore, the routinely use of MB staining in PSD surgery should be considered.

## 1. Introduction

Pilonidal Sinus Disease (PSD) presents as a debilitating skin infection in the coccyx area, with increasing global incidence [1], imposing significant distress on affected individuals. Its etiology, while not entirely understood, is associated with several risk factors, such as increased hairiness in the region, male gender, and family history [2,3,4]. Predominantly considered an acquired condition, PSD is believed to initiate from occipital hair fragments [5] migrating down the spine [6] to penetrate the skin, leading to foreign body reactions, partial fistula tract epithelialization, and chronic infection [6]. However, with up to fifty percent of pathological specimens lacking hair, the disease’s multifactorial etiology remains elusive, with certain families displaying higher incidences, suggesting a possible genetic predisposition [7].

Historically, treatments have ranged from X-rays [8] to laser ablations [9,10,11], yet surgical intervention remains the sole curative strategy. Various surgical techniques have evolved, each offering distinct benefits and limitations. Specifically, techniques that leave the suture off-midline and alter the sacral area’s surface contour above the rima ani—through either advancing (e.g., Karydakis [12] or Bascoms Cleft-lift [13]) or pivoting flaps (e.g., Limberg [14,15] or Dufourmentel [16])—demonstrate notably lower PSD recurrence rates [17]. The main goal of PSD surgery is the complete excision of the infected areas and all fistulas. The hypothesis that unexcised fistula tracts contribute to recurrent PSD has spurred additional methods to enhance tract identification, including probing [18,19] and intra-operative dyes like methylene blue (MB). Dyeing the tracts aids in the meticulous identification of subsidiary ducts, facilitating more comprehensive surgical excision [20,21,22] and reducing recurrence risks by up to 50% [23]. The use of MB not only aids in identifying even the most minuscule fistulas—crucial for less experienced surgeons—but also offers antimicrobial benefits that may enhance treatment efficacy [24,25].

Prompted by these considerations, our study embarks on an extensive analysis of the existing literature to assess the impact of MB on PSD surgery recurrence rates globally.

## 2. Methods

**Figure 1:**
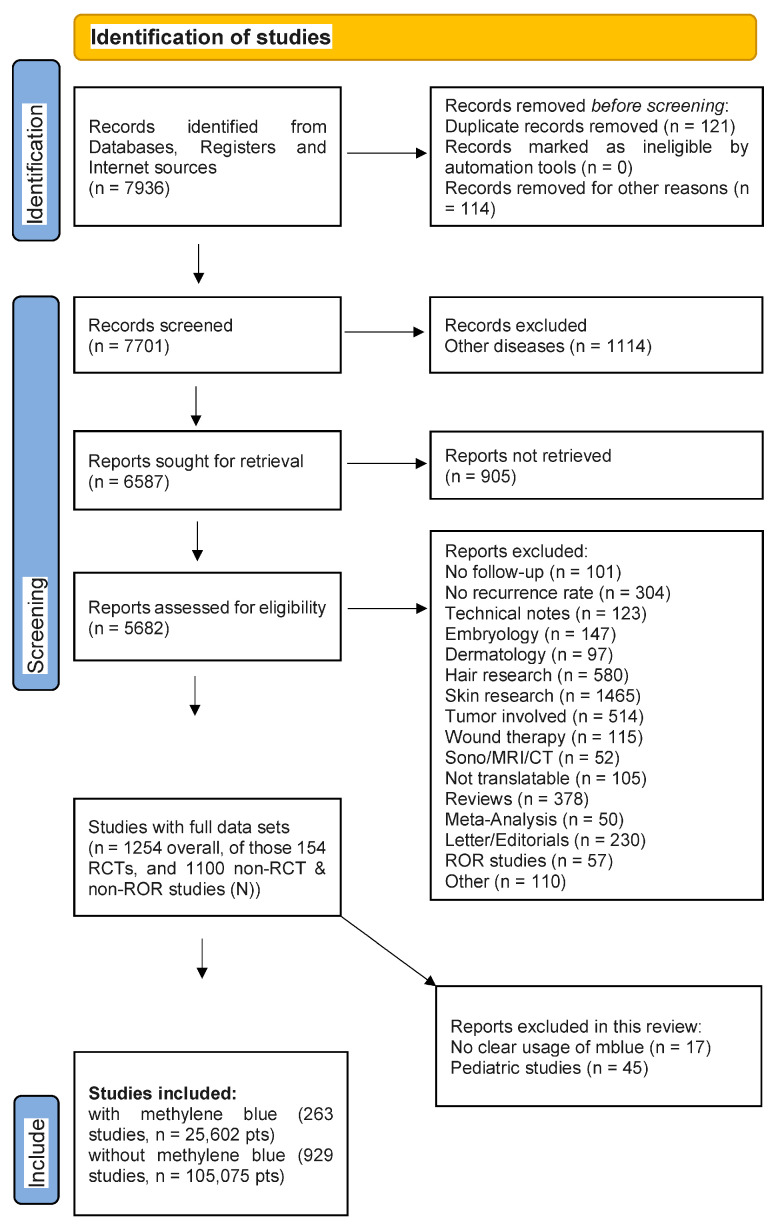
PRISMA flow chart of study harvest and qualification steps.

The data retrieval process adhered to the Stauffer methodology [26] and yielded the results depicted in the PRISMA flow diagram (Figure 1).

### 2.1. Search Strategy and Study Selection Criteria

This systematic review was conducted in accordance with the Preferred Reporting Items for Systematic Reviews and Meta-Analyses (PRISMA) 2020 statement [27]. The completed PRISMA 2020 checklist is provided as Supplementary Material S1. A formal protocol was not prospectively registered in PROSPERO given the historical and scoping nature of the data synthesis. However, the study adhered to a predefined internal protocol covering search strategy, eligibility criteria, and analysis plans, as detailed below.

To compile an exhaustive database on Pilonidal Sinus Disease (PSD), we conducted a systematic review of the literature using the NCBI Medical Subject Heading (MeSH) term “pilonid*” along with a combination of “dermoid” and “cyst.” Our search spanned multiple databases: MEDLINE, PubMed, PubMed Central, Scopus, Ovid, Embase, and the Cochrane Central Register of Controlled Trials (CENTRAL). Additionally, we extended our search to Google, Google Scholar, and ResearchGate and critically reviewed references cited in both national and international guidelines, including the S3 guidelines from the Association of the Scientific Medical Societies in Germany related to PSD treatment. We also evaluated the bibliographies of all acquired documents. The scope of documents reviewed included a range of study designs—randomized (RCT) and non-randomized trials (nRCT), prospective and retrospective studies, and observational studies such as cohort studies, case-control studies, cross-sectional studies, and case reports, covering the period from 1833 to 2023. Even though surgical techniques and general medical treatments have evolved remarkably over this period, we analyzed all of the available literature from this timeframe rather than focusing solely on the last few decades. This approach was necessary because the use of MB is not common practice, and we aimed to compile a comprehensive dataset. The search window spanning 1833 to 2023 was intentionally broad to provide a comprehensive overview of the surgical evolution of Pilonidal Sinus Disease (PSD) treatment. Inclusion of the pre-antibiotic and historical literature was deemed necessary to capture the widest possible range of outcomes for MB staining, a technique that has not been common practice in recent decades but offers significant visualization benefits. This longitudinal approach allows for the assessment of surgical principles that transcend specific medical eras.

Four authors (TH, MB, MBM, DD) rigorously assessed the retrieved documents against the inclusion criteria. These criteria required details on definitive treatment, the use of MB in surgery, recurrence rates, and the duration of follow-up. We considered reports published in English, French, German, Italian, and Spanish, and also those in other languages, provided they included an English abstract with relevant details. When necessary, authors were contacted for translations via email or ResearchGate.

Our exclusion criteria were Pilonidal disease outside the presacral region, the involvement of neoplastic conditions, and duplicate data publication by the same author. We excluded studies lacking any element of the minimal data set, which includes information on definitive treatment strategy, recurrence, and follow-up duration. While we excluded previous meta-analyses and review articles, we thoroughly examined their reference lists for additional evidence. Unpublished data cited in review articles were considered. Studies that determined recurrence solely based on the principle of “return on recurrence”, without proactively investigating the majority of patients who did not return, were also excluded due to known observation bias.

### 2.2. Data Collection, Extraction, and Quality Assessment

We conducted a comprehensive analysis and meticulously documented all identified studies, culminating in a cross-study data synthesis. The extracted data were carefully put into a Microsoft Excel spreadsheet (Version 2016, Microsoft Corp., Redmond, WA, USA) for subsequent verification to guarantee accuracy. Each unique therapeutic approach identified in the studies was assigned a dedicated row. The spreadsheet columns included citation details, the number of patients involved, descriptions of therapeutic procedures, reported follow-up durations, study specifics, and recurrence rates. The methodological quality and risk of bias of the included studies were independently assessed by two reviewers (TH and MM). Owing to the wide heterogeneity of study designs and the extended historical period covered (1833–2023), no single validated risk-of-bias tool was applicable across all included publications. Therefore, a structured qualitative assessment was performed using predefined methodological criteria. Quality assessment focused on the clarity of patient selection, transparency and reproducibility of the surgical technique described, adequacy and consistency of outcome definitions, completeness and reliability of follow-up reporting, and the plausibility of recurrence ascertainment. Based on these criteria, studies were categorized as having high, moderate, or low methodological quality. Discrepancies in quality assessment between the two reviewers were resolved through discussion and consensus with a third author (DD). The use of two independent reviewers (TH and MM) for quality assessment, with resolution of discrepancies via a third senior author (DD), was a core component of our strategy to minimize reviewer bias and ensure the integrity of the qualitative synthesis. Due to the variability in statistical measures for reporting follow-up times across the studies, we treated mean and median times as comparable. This approach was justified by the predominance of disease occurrence in young adults. In instances where only minimum follow-up times were available, these values were duly recorded as provided.

Due to the retrospective nature of this cross-study synthesis, covering literature from 1833 to 2023, the definition of “recurrence” was dictated by the primary sources. To ensure maximum consistency, we primarily extracted data where recurrence was defined as the clinical return of symptoms or the necessity for re-intervention. Studies using a passive “return on recurrence” (ROR) model were excluded to minimize observation bias. While the variability of these definitions is a known limitation of large-scale historical meta-analyses, the inclusion of “Number at Risk” tables provides the necessary transparency regarding follow-up reliability and censoring patterns.

### 2.3. Surgical Procedures Analyzed and Statistical Methodology

The surgical procedures evaluated in this study include the following:

1. Primary Open Approach: In this method, the Pilonidal Sinus Disease (PSD) is resected, and the wound is left open, allowing it to close gradually through secondary healing.
2. Primary Midline Closure: This technique involves suturing the wound borders along the midline of the anal cleft, resulting in a midline scar.
3. Primary Asymmetric Closure: Here, the wound borders are sutured outside of the midline, offering an alternative to midline closure (advancement flap).
4. Bascom and Karydakis Techniques: Both techniques involve mobilizing one side of the wound to achieve an off-midline closure advancement flap. Due to their similar approaches, they were analyzed as a combined group.
5. Limberg and Dufourmentel Rhomboid Flap Techniques: These methods use a rotated skin flap to close the wound off the midline and were grouped together because of their comparable techniques.
6. Other Flap Techniques: Various other flap techniques designed to achieve tension-free wound closure were also reviewed.

To ensure that these categories were mutually exclusive, each study was assigned to a single group based on the primary surgical intent described in the original text; hybrid procedures or those with ambiguous surgical descriptions were excluded from the subgroup analysis to maintain the integrity of the taxonomy. Furthermore, this classification allows for a distinct comparison between midline and off-midline procedures, effectively isolating technique-level recurrence risks as a potential confounder. The therapeutic procedures were further categorized based on the use of MB during surgery.

Statistical analysis and data visualization were performed using R statistical software (version 4.3.2) within the RStudio framework (version 23.6.1.524). A significance level was set at *p* < 0.05, and analyses were conducted using a two-tailed approach. Kaplan–Meier survival analysis, complemented by pointwise 95% confidence intervals (CIs), was utilized to assess recurrence-free outcomes over time, using the “survival” package (version 3.5.7) in R. Graphical representations were generated with the “survminer” package (version 0.4.9).

The results were presented as percentages of recurrence-free outcomes alongside their respective 95% CIs. The horizontal axes of the plots indicated the number of patients within specific follow-up periods, measured in months. For intervals lacking specific data, linear interpolation was applied to estimate recurrence-free outcomes based on the nearest observed follow-up times.

To ensure a comprehensive evaluation of recurrence and to provide a robust foundation for treatment selection, the study analyzed data akin to a classical meta-analysis of RCTs and also incorporated analyses of non-RCTs. Since no RCTs exist on the use of MB and recurrence rates, a meta-analysis was not feasible. Additionally, forest plots were not used, as they do not allow for the visualization of the time dependency of recurrence rates (RRs), which is one of the major influencing factors for RR. Due to the incremental nature of the Kaplan–Meier curves, slight discrepancies between plotted and tabulated values might be observed.

To ensure a robust foundation for analysis across heterogeneous study designs, we utilized the methodology described by Tierney et al. to calculate Hazard Ratios (HRs) and their 95% Confidence Intervals (CIs). For studies where only crude recurrence proportions and follow-up durations were provided, time-to-event data were derived based on the assumption of a constant hazard rate within the reported follow-up period. For studies providing Kaplan–Meier curves, these were digitized to extract individual patient data approximations. These study-level estimates were then aggregated to generate the pooled KM curves. This approach allowed for the visualization of time-dependent recurrence rates, which is a major influencing factor in Pilonidal Sinus Disease (PSD) outcomes.

The sheer amount of literature sources found in the world literature exceeds being appended in full. A detailed literature appendix is available on request to the corresponding author.

## 3. Results

Our analysis included 1192 studies involving 130,677 patients. A total of 263 studies were performed with MB (*n* = 25,602 patients), and 929 studies did not include MB (*n* = 105,075 patients). From these studies, we analyzed the different surgical procedures concerning their rate of recurrence.

### 3.1. Overall Study Population

**Figure 2:**
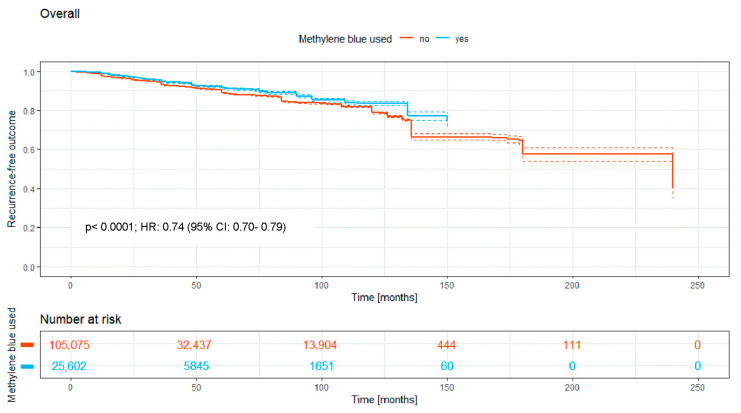
Kaplan–Meier curve of all analyzed studies, comparing usage of MB staining (yes = blue curve) and surgery without MB (no = red curve).

In total, 130,677 patients received surgery; 25,602 patients (19.6%) received MB staining, and 105,075 patients (80.4%) received no MB staining. The Kaplan–Meier curve (Figure 2) shows the recurrence-free outcome of the two groups over a time period of a maximum of 240 months. There is a significant difference between both curves, with a *p*-value of <0.0001.

### 3.2. Primary Open Approach

**Figure 3:**
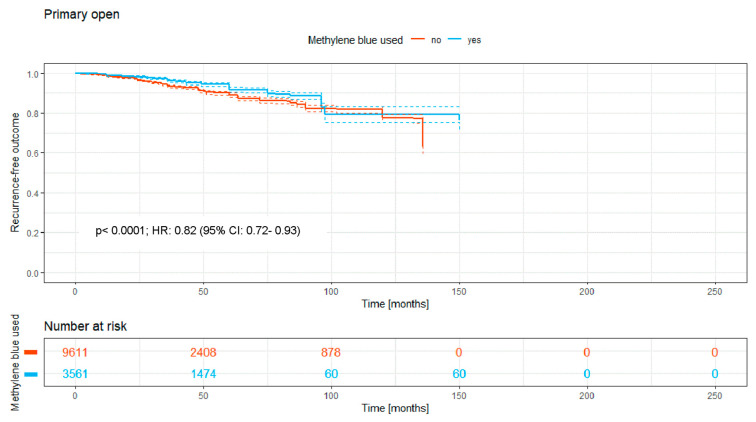
Kaplan–Meier curve of all analyzed studies with primary open approach, comparing usage of MB (yes = blue curve) and surgery without MB (no = red curve).

The number of patients treated with the traditional open approach was 13,172. Of these, 3561 patients (27%) received MB staining during surgery, and 9611 patients (73%) were operated on without MB. The Kaplan–Meier curve (Figure 3) shows the recurrence-free outcome of the two groups over a time period of a maximum of 150 months. There is a significant difference between both RR curves, with a *p*-value of <0.0001.

### 3.3. Primary Midline Closure

**Figure 4:**
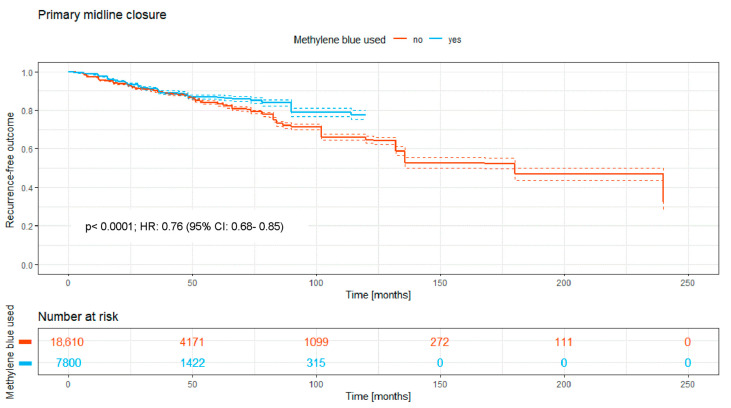
Kaplan–Meier curve of all analyzed studies with primary midline closure approach, comparing usage of MB staining (yes = blue curve) and surgery without MB (no = red curve).

A total of 26,410 patients were treated with primary midline closure; 18,610 patients (70.5%) received surgery without MB staining and 7800 (29.5%) with MB staining. The Kaplan–Meier curve (Figure 4) shows the recurrence-free outcome of the two groups over a time period of a maximum of 240 months. There is a significant difference between both RR curves, with a *p*-value of <0.0001.

### 3.4. Primary Asymmetric Closure

There were just 3357 patients who received a primary asymmetric closure; of these, 1347 (40.1%) patients received MB staining and 2010 (59.9%) did not.

**Figure 5:**
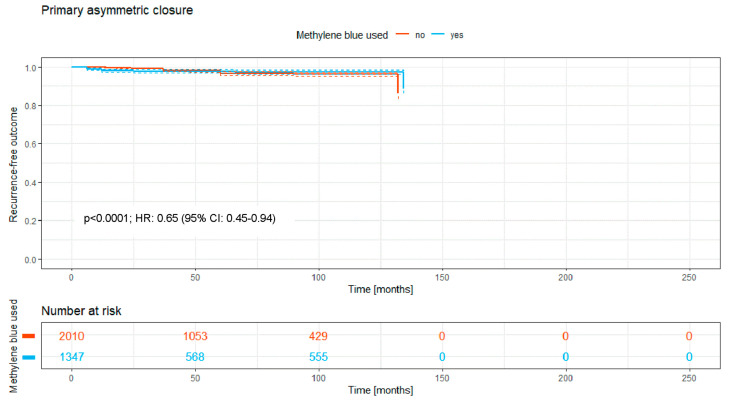
Kaplan–Meier curve of all analyzed studies with primary asymmetric closure, comparing usage of MB staining (yes = blue curve) and surgery without MB (no = red curve).

The Kaplan–Meier curve (Figure 5) shows the recurrence-free outcome of the two groups over a time period of a maximum of 140 months. There is a significant difference between both RR curves, with a *p*-value of <0.0001.

### 3.5. Bascom and Karydakis (Lateral Advancement Flap Technique)

The group with the lateral closure techniques of Bascom and Karydakis consisted of 21,254 patients. Just 2615 (12.3%) received MB staining during surgery, with 87.7% of patients (*n* = 18,639) not receiving staining.

**Figure 6:**
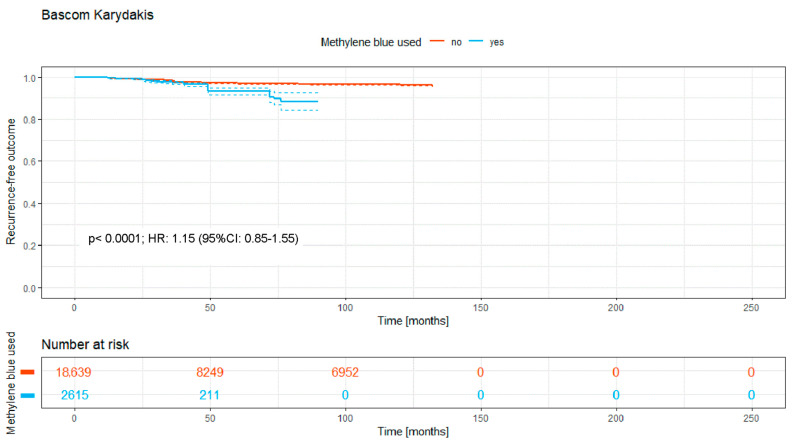
Kaplan–Meier curve of all analyzed studies with the Bascom or Karydakis closure technique, comparing usage of MB staining (yes = blue curve) and surgery without MB (no = red curve).

The Kaplan–Meier curve (Figure 6) shows the recurrence-free outcome of the two groups over a time period of a maximum of 130 months.

There is a significant difference between both curves, with a *p*-value of <0.0001, showing a significant disadvantage for the stained group. Notably, after 50 months, just 211 patients were left in the MB follow-up group, rendering the *p*-value not meaningful, as shown with an HR of 1.15.

### 3.6. Limberg and Dufourmentel (Rhomboid Rotation Flap Technique)

The flap-closure techniques of Limberg or Dufourmentel were used for 18,654 patients in the studies analyzed. The majority, 64.4%, did not receive a MB staining (*n* = 12,017 patients), with 6637 patients (35.6%) treated with MB.

**Figure 7:**
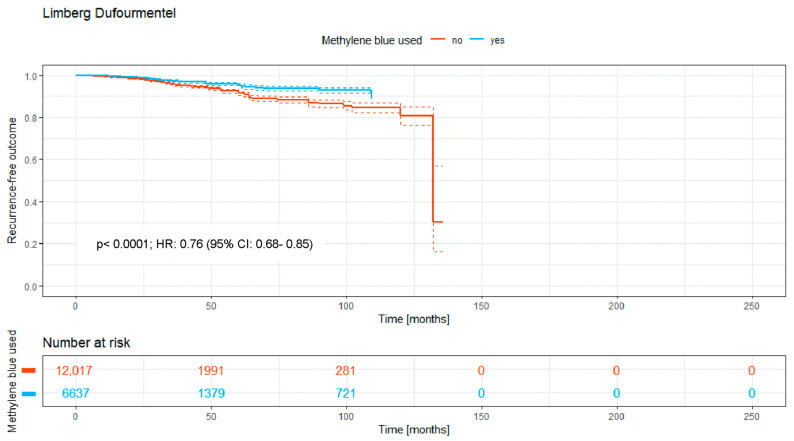
Kaplan–Meier curve of all analyzed studies with Limberg or Dufourmentel flap technique, comparing usage of MB staining (yes = blue curve) and surgery without MB (no = red curve).

The Kaplan–Meier curve (Figure 7) shows the recurrence-free outcome of the two groups over a time period of a maximum of 130 months. There is a significant difference between both curves, with a *p*-value of <0.0001.

### 3.7. Other Flap Techniques

There were 5287 patients treated with other flap techniques, of whom 2021 (38.2%) received MB staining and 3266 (61.8%) were operated on without MB.

**Figure 8:**
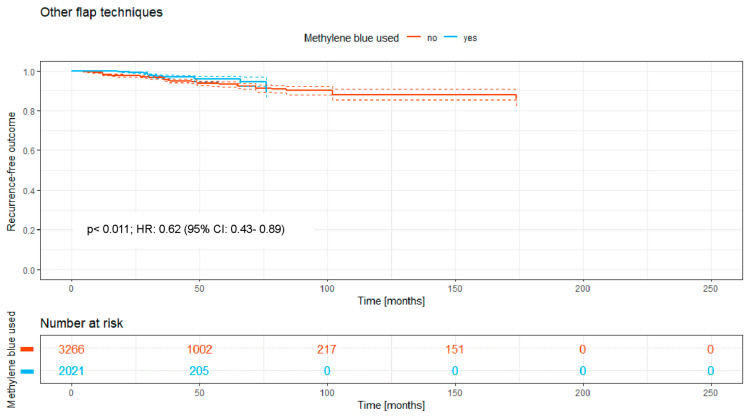
Kaplan–Meier curve of all analyzed studies with other flap techniques, comparing usage of MB staining (yes = blue curve) and surgery without MB (no = red curve).

The Kaplan–Meier curve (Figure 8) shows the recurrence-free outcome of the two groups over a time period of a maximum of 170 months. There is a significant difference between both curves, with a *p*-value of 0.011, with a significant disadvantage for the unstained group. Notably, after 50 months, just 205 patients were left in the blue follow-up group, diminishing the meaning of the *p*-value.

### 3.8. Five-Year Recurrence Rate

To achieve an optimized comparability between the different treatment groups due to dissimilar follow-up periods, a cut-off focus was set for a 5-year (60-month) recurrence follow-up (Table 1). The treatment groups with the primary open approach, primary midline closure, primary asymmetric closure, and Limberg/Dufourmentel all showed a significantly diminished rate of recurrence with MB dye (*p* < 0.0001). In detail, within 5 years the primary open approach showed 10.9% RR without MB as opposed to 8.3% with MB, primary midline closure showed 16.8% without MB and 13.4% with MB, primary asymmetric closure showed 3.5% and 2.3%, and Limberg/Dufourmentel resulted in 8.6% and 4.8%, respectively. The only outliers were the two groups of Bascom/Karydakis and Other Flaps. While the second group had a slightly lower significance (*p* = 0.011), showing a recurrence rate of 6.7% without versus 4.2% with MB staining, Bascom/Karydakis showed an inverse relationship, with a higher recurrence rate of 6.8% with MB staining versus 3.1% without. Both groups had a high dropout rate at follow-up, which limits the significance of the *p*-values. The total number of examinations resulted in a recurrence rate of 11% in 130,677 treated patients without MB, while the use of MB reduced the recurrence rate to 8.6% (*p* < 0.0001).

Overall, the intraoperative use of MB was associated with a significantly higher recurrence-free survival rate (HR: 0.74; 95% CI: 0.70–0.79; *p* < 0.0001; Figure 2). Significant benefits were observed across subgroups, including the primary open approach (*p* < 0.0001; HR: 0.82 [95% CI: 0.72–0.93]), primary midline closure (*p* < 0.0001; HR: 0.76 [95% CI: 0.68–0.85]), and primary asymmetric closure (*p* < 0.0001; HR: 0.65 [95% CI: 0.45–0.94]). The most pronounced benefit was noted in the Limberg/Dufourmentel subgroup (HR: 0.55; 95% CI: 0.42–0.72; *p* < 0.0001; Figure 7). In contrast, the Bascom/Karydakis group showed an inverse trend (HR: 1.15; 95% CI: 0.85–1.55). However despite the Log-rank *p* value (*p* < 0.0001), the Hazard Ratio did not reach statistical significance, as the confidence interval includes 1.00. This discrepancy, along with the significant loss to follow-up (only 211 patients at risk after 50 months), suggests that the observed data in this subgroup are insufficient to draw definitive conclusions regarding an advantage for MB (Figure 6).

## 4. Discussion

The challenge of recurrences post-pilonidal sinus surgery significantly impacts patients and surgeons. The literature identifies inadequate excision as a pivotal etiological factor in PSD recurrence [28]. Consequently, more extensive operations, which are linked to a heightened risk of wound-healing disorders, suggest that merely increasing radicality does not constitute the best approach for PSD management.

The long-practiced technique of employing MB [20,22,29,30,31] for enhanced visualization of fistula tracts facilitates the safe resection of even the finest side ducts. Whether this technique has a beneficial effect on the recurrence rate in PSD surgery was not focus of prior research. With ongoing discussions about the benefits of MB staining, we analyzed 1192 studies to identify a significant link between MB use and reduced recurrence rates in PSD surgery. Despite lower case numbers and follow-up data in MB studies potentially influencing statistics, our findings are promising.

The overall data (Figure 2) indicate a lower 5-year recurrence rate in PSD surgery with MB dye usage (8.6%) compared to without (11%), suggesting MB’s efficacy. This significant reduction in recurrence rates presents a compelling case for incorporating MB.

Upon analyzing grouped data and Kaplan–Meier curves, a critical perspective is still necessary despite generally convincing outcomes. The primary open approach still is the most commonly used surgical procedure in PSD surgery worldwide [1]. Our data show that if the resection takes place without closure of the wound, the prior use of MB dye decreases the risk of recurrence significantly to 8.3% compared to resection without MB (10.9%). This technique shows less risk of recurrence than the primary midline closure, one of the reasons why the latter technique is considered obsolete. Still, even when considering this to be abandoned technique [1], the use of MB dye facilitates a decrease of the recurrence risk from 16.8% without MB to 13.4% with MB, presenting a considerably high risk but a statistically significant decrease. The primary asymmetric closure of the wound shows very closely aligned Kaplan–Meier curves (Figure 4). But even with this rather small sample size, the use of MB results in statistical significance regarding recurrence rates.

Grouping the (modified) Limberg and Dufourmentel flaps for analysis due to their similarities does make sense. The findings revealed that the use of MB dye significantly reduced the recurrence rate to 6.8% compared to 8.6% without MB over five years, underscoring MB’s potential benefit in PSD surgery outcomes (Figure 6). The statistical analysis for both the Bascom/Karydakis and other flap techniques requires careful consideration due to significant follow-up loss after 50 months. This drop in follow-up participants to 8% and 10%, respectively, impacts the reliability of the 5-year recurrence rate’s statistical significance. Specifically, for Bascom/Karydakis, an observed disadvantage in recurrence rates for the group treated with MB (MB) dye is notable but must be critically evaluated due to the low follow-up rate. This caution also applies to interpreting the statistical significance of outcomes in the “other flaps” group. The inverse relationship observed in the Bascom/Karydakis subgroup warrants a mechanical and statistical interpretation. Clinically, a potential mechanism for higher recurrence when using MB in advancement flaps is the misclassification of dye extravasation as a tract extension. If dye is injected under high pressure, it may stain healthy surrounding tissue, leading to a more radical resection than necessary. In procedures that rely on tension-free mobilization, such as the Bascom or Karydakis techniques, increased resection depth can lead to heightened wound tension, a known risk factor for recurrence. Statistically, the significant loss to follow-up in the MB arm—dropping to only 211 patients at risk after 50 months—suggests that these late-stage findings are highly susceptible to attrition bias and should not be viewed as a definitive contraindication for MB in these techniques.

When comparing these findings to the existing literature, it is apparent that few studies have analyzed MB application in PSD surgery. Doll et al. (2008) [23] explored the use of MB for dyeing fistular tracts prior to surgery in a cohort of 247 patients, revealing that its application significantly reduced the recurrence rate from 30% in untreated patients to 16% in treated patients over an average follow-up of 14.9 years. Notably, chronic PSD patients treated with MB exhibited a 19% recurrence rate, compared to 24% in untreated cases, while acute PSD patients showed a recurrence rate of 19% with MB treatment versus 43% without, suggesting a nuanced effect of MB based on PSD’s acute or chronic status. The precise mechanisms —whether antibacterial properties or improved surgical visibility— warrant further investigation. Idiz et al. (2014) [32] adopted a critical stance, evaluating MB’s effectiveness in preventing incomplete excisions in sacrococcygeal PSD surgery through a prospective RCT. Their findings cautioned against reliance on MB marking alone due to potentially insufficient resections, thereby raising concerns about increased recurrence risks. Conversely, Ardelt et al. (2016) [33] and Durgut (2018) [34] provided contrasting evidence regarding the impact of MB on the volume of resected specimens, with Durgut reporting significantly smaller dimensions in excised specimens, except for resection depth, highlighting the need for a nuanced application of MB.

The impact of MB dye on reducing recurrence rates in PSD surgery may involve more than its role in improving visibility. Its historical use, noted by Paul Ehrlich in 1881 [35] for its antimicrobial properties, suggests MB could reduce bacterial content in the sinus tracts, potentially contributing to surgical success. The update of the German S3 guideline on the treatment of PSD does mention the use and advantage of MB in the surgical treatment of PSD [1]. Due to the classification of MB into the Acute Tox 4 category (new European Union regulations), the application should be reconsidered, and alternatives such as toluidine blue or patent blue should be examined. It remains to be determined whether the well-known antimicrobial properties of MB justify the risk of use. As new study results have not yet been published, no recommendation for their use is currently available [1]. Should future studies with Toluidine-blue as a dye produce less effective results concerning recurrence than MB, then the aforementioned antibacterial properties might be more important than are currently estimated.

Given the large sample size of 130,677 patients, the high statistical significance (*p* < 0.0001) observed in our results must be interpreted alongside its clinical relevance. In our total population, the use of MB staining reduced the recurrence rate from 11% to 8.6%. While a difference of 2.4 percentage points may appear small, it represents a Relative Risk Reduction (RRR) of 21.8%. To put this into clinical perspective, the Number Needed to Treat (NNT) is approximately 42, meaning that staining the fistula tracts in 42 patients prevents one additional recurrence. Considering that MB staining is a low-cost, low-risk, and minimally invasive intervention, this absolute reduction constitutes a clinically significant improvement in surgical outcomes.

This study has certain limitations. Recognizing the constraints of retrospective analyses is essential, as they inherently pose challenges that a prospective, multicenter, randomized study could mitigate by providing more robust data. Previous studies have suggested that analyzing the 5-year recurrence rate in PSD surgery may be insufficient to fully capture all relevant differences in recurrence rates (RRs). The longer the follow-up period, the greater the number of recurrences observed, with more reliable data emerging after 10 to 20 years.

Furthermore, incorporating published data spanning nearly 190 years presents certain risks. Over this period, surgical techniques, antibiotic regimens, anesthesia methods, and broader life-altering events—such as two world wars and two pandemics—have undoubtedly influenced medical practices. While a sensitivity analysis restricted to papers after 2000 was considered, we believe that the inclusion of all available data provides the most robust evidence for the fundamental surgical principle of visualization. Notably, we performed a focused “modern-era” sensitivity analysis by isolating post-2000 off-midline procedures with a documented follow-up of at least 24 months. The significant benefits observed in these modern flap techniques—such as in the Limberg/Dufourmentel group—suggest that the efficacy of MB is not a relic of older, more radical surgical eras but remains highly relevant in contemporary practice. By stratifying our analysis according to surgical taxonomy (Figure 3, Figure 4, Figure 5, Figure 6, Figure 7 and Figure 8), we have partially mitigated confounding by indication, as MB showed a consistent protective effect across diverse procedural categories. Although variables such as surgeon learning curves and center volume could not be adjusted for via meta-regression due to inconsistent reporting in historical sources, the consistency of the Hazard Ratios across subgroups suggests a robust underlying biological and surgical effect of MB staining. However, the inclusion of all existing studies introduces additional limitations. A cross-study data synthesis necessitates extracting data from existing publications while accepting the data as reported. Variability in study quality, methodological heterogeneity, and potential biases—such as those related to study design, allocation, blinding, and selective reporting—may influence the collective data. These factors could, in turn, affect the reliability of the synthesis and potentially weaken the final conclusions drawn from our analysis.

Future research should leverage Large Language Models (LLMs) and Artificial Intelligence (AI), which have already shown promise in dermatology for inflammatory skin conditions [36]. Analogous AI-assisted tools could standardize PSD phenotype labeling in operative notes—specifically distinguishing primary open, midline, and off-midline flap types—while capturing tract complexity and prompting minimum operative checklists (e.g., MB concentration and injection method). Furthermore, computer-vision approaches using standardized cleft photography could quantify healing trajectories and flag early recurrence. Such advancements would bridge historical surgical principles with modern data governance and predictive analytics, creating personalized risk tiers based on baseline complexity and operative variables.

While technical reporting varied significantly across the analyzed studies, our synthesis suggests that a standardized application protocol is essential for surgical reproducibility. We recommend the use of 0.5% to 1% methylene blue (MB) diluted in NaCl or hydrogen peroxide. The dye should be injected prior to the start of excision directly into the external pits or the sinus cavity. A total volume of 0.5 to 1 mL is typically sufficient to visualize the fistula tracts without causing high-pressure extravasation into surrounding healthy tissue, which can hinder surgical precision. This standardized approach facilitates the meticulous identification of subsidiary ducts while minimizing the risk of over-resection. Despite its benefits, the use of MB must be balanced against its safety profile. Although not systematically reported in the historical data synthesized here, potential local adverse events include local pain and tissue tattooing that may interfere with histopathological margin assessment [37]. Systemic risks, while rare in the low doses used for staining, include methemoglobinemia and serotonin syndrome [38], particularly in patients on serotonergic antidepressants. Furthermore, MB should be avoided in patients with glucose-6-phosphate dehydrogenase (G6PD) deficiency [39]. This safety checklist is particularly relevant, as modern regulations prompt a transition toward potential alternatives like toluidine blue.

## 5. Conclusions

Our analysis of 130,677 patients demonstrates that a simple injection of methylene blue (MB) at the start of PSD surgery significantly reduces the 5-year recurrence rate from 11% to 8.6%. While the massive sample size ensures high statistical significance, the clinical impact is equally substantial, reflected in a Relative Risk Reduction (RRR) of 21.8% and a Number Needed to Treat (NNT) of 42. This benefit remains consistent across various surgical techniques, including modern flap procedures. Given its low cost and ease of implementation, MB staining offers a favorable cost–benefit ratio and should be considered a standard practice to optimize fistula tract visibility. Future integration of AI and LLM-based tools may further standardize these protocols and provide personalized risk stratification.

## Figures and Tables

**Table 1:** Five-year recurrence rates of the treated patients in the different treatment groups (row 1), with the number of treated patients (*n*) and the recurrence rate in % with MB staining (MB+) and without MB staining (MB−) as well as the *p*-value at the 60-month mark (* the significance of the *p*-value is limited due to the high drop-out rates at follow-up after 50 months).

5-Year Recurrence Rate	Patients [*n*]	MB+ [%]	MB− [%]	*p*-Value
Overall	130,677	8.6	11	<0.0001
Primary open	13,172	8.3	10.9	<0.0001
Primary midline closure	26,410	13.4	16.8	<0.0001
Primary asymmetric closure	3357	2.3	3.5	<0.0001
Bascom Karydakis	21,254	6.8	3.1	<0.0001 *
Limberg Dufourmentel	18,654	4.8	8.6	<0.0001
Other flaps	5287	4.2	6.7	0.011 *

## Data Availability

The datasets used and analyzed during the current study are available from the corresponding author on reasonable request. The data are not publicly available at this time to protect the integrity of ongoing longitudinal analyses and further research projects based on this comprehensive database.

## Supplementary Materials

The following supporting information can be downloaded at: https://www.mdpi.com/article/10.3390/medicina62020238/s1, PRISMA 2020 Checklist.

## Abbreviations

RR, recurrence rate; ROR, return on recurrence; PSD, Pilonidal Sinus Disease; MB, methylene blue.
